# Radiotherapy outcomes and risk factors for young patients with head-and-neck squamous cell carcinomas: a matched-pair analysis

**DOI:** 10.1186/s13014-025-02631-w

**Published:** 2025-04-22

**Authors:** Jiadai Zou, Alexander Rühle, Henning Schäfer, Andreas Dietz, Gunnar Wichmann, Thomas Kuhnt, Anca-L. Grosu, Nils H. Nicolay

**Affiliations:** 1 Department of Radiation Oncology, University of Leipzig Medical Center, Stephanstr. 9a, 04103 Leipzig, Germany; 2 Comprehensive Cancer Center Central Germany, Partner Site Leipzig, Leipzig, Germany; 3 Department of Radiation Oncology, University Medical Center Freiburg, Freiburg, Germany; 4 German Cancer Consortium (DKTK), Partner Site Freiburg, German Cancer Research Center, Heidelberg, Germany; 5 Department of Otorhinolaryngology, University of Leipzig Medical Center, Leipzig, Germany

**Keywords:** Radiotherapy, Chemotherapy, HNSCC, Head-and-neck cancer, Young

## Abstract

**Background:**

Head-and-neck squamous cell carcinomas (HNSCC) exhibit significant variations in incidence and outcomes across age groups. There is conflicting data on the oncological outcomes of younger HNSCC patients ≤ 45 years. This study analyzed clinical characteristics, treatment-related toxicities and survival rates of young HNSCC patients treated with (chemo)radiotherapy.

**Methods:**

HNSCC patients ≤ 45 years treated with radiotherapy between 2009 and 2021 at two large cancer centers were analyzed and matched to a patient cohort > 45 years based on TNM and tumor localization. Overall (OS), progression-free (PFS) and metastasis-free (DMFS) survival and locoregional control (LRC) were compared and treatment-related toxicities were assessed.

**Results:**

99 patients were included in this analysis. Median OS of the young HNSCC cohort was 63 months. Daily alcohol consumption was identified as a key risk factor for reduced OS in the multivariate analysis. OS was similar in the young cohort compared to older patients, although the excess mortality risk compared to the sex- and age-matched general population amounted to 59-fold, while it was only 5.9-fold for patients ≥ 45 years. No significant differences were observed in PFS, LRC, or DMFS between age groups. Higher-grade chronic toxicities were moderate in young HNSCC patients.

**Conclusions:**

Young HNSCC patients ≤ 45 years treated with (chemo)radiation have similar rates of oncological survival outcomes compared to older patients. While chronic toxicities from (chemo)radiation are low, further research is needed to explore the long-term quality-of-life.

**Supplementary Information:**

The online version contains supplementary material available at 10.1186/s13014-025-02631-w.

## Introduction

Head-and-neck squamous cell carcinomas (HNSCC) account for approximately 5% of all new cancer diagnoses globally, and 900,000 new cases and 450,000 deaths per year have been reported by the latest GLOBOCAN estimates [1]. The average age at diagnosis is 64 years with only a small minority of patients diagnosed at a younger age [2]. The age cohort of so-called “young” patients has not yet been well-defined for HNSCC, and most reports use an age cut-off between 40 and 50 years [3]. The most common localizations of HNSCC in young patients are the oral cavity, especially the tongue and the tonsils, and it has been suggested that for those localizations, there is an increased cancer incidence at a young age [4, 5]. Based on differences in etiology and risk factors, it has been hypothesized that HNSCC biology varies between age groups: While the exposure to established risk factors such as smoking or alcohol is less likely and of shorter duration in young HNSCC patients, these patients are more often affected by human papilloma virus (HPV)-driven cancers [6–8]. On the other hand, it has been widely suggested that HNSCC at a younger age is characterized by a more aggressive tumor biology and an association with familial cancer histories [9–12]. Genetic predispositions and inherited syndromes like Fanconi Anemia and Bloom’s syndrome may also contribute to HNSCC development in young adults [13, 14].

Incidence rates of young HNSCC patients considerably vary between individual regions, and while low rates of 3 to 6% patients diagnosed at age 45 or younger have been reported for Europe and North America, the percentage has been calculated at 17.2% in Africa and 14.5% in the Middle East [3, 15].

Available data on the outcome of patients ≤ 45 years with HNSCC are scarce and conflicting, and most available reports are retrospective single-center analyses and mix limited stages amenable to single-modality treatment (often surgical resection) and locally or regionally advanced stages requiring multi-modal treatment. In this context, several publications have shown an improved overall survival (OS) of young patients compared to their older counterparts despite comparable rates of disease-specific survival [9, 16–18]. While some studies report a higher incidence of node-positive disease in young HNSCC patients at presentation, the correlation between age and oncological survival outcomes due to metachronous regional or distant metastases remains unclear [17, 18]. The choice of optimal treatment for improving outcomes in young patients with locoregionally advanced HNSCC remains somewhat unclear, but regarding the real-world clinical situation, publications suggest that upfront surgical approaches with the addition of chemotherapy to radiotherapy in the adjuvant setting are more commonly employed [19, 20].

Here, we report demographic data, clinical outcomes and treatment-related toxicities in young patients ≤ 45 years treated between 2009 and 2021 at two large tertiary cancer centers in Germany. Additionally, we compared survival and locoregional control rates of young patients with those of patients > 45 years based on a matched-pair analysis.

## Methods

### Patient cohort

**Fig. 1:**
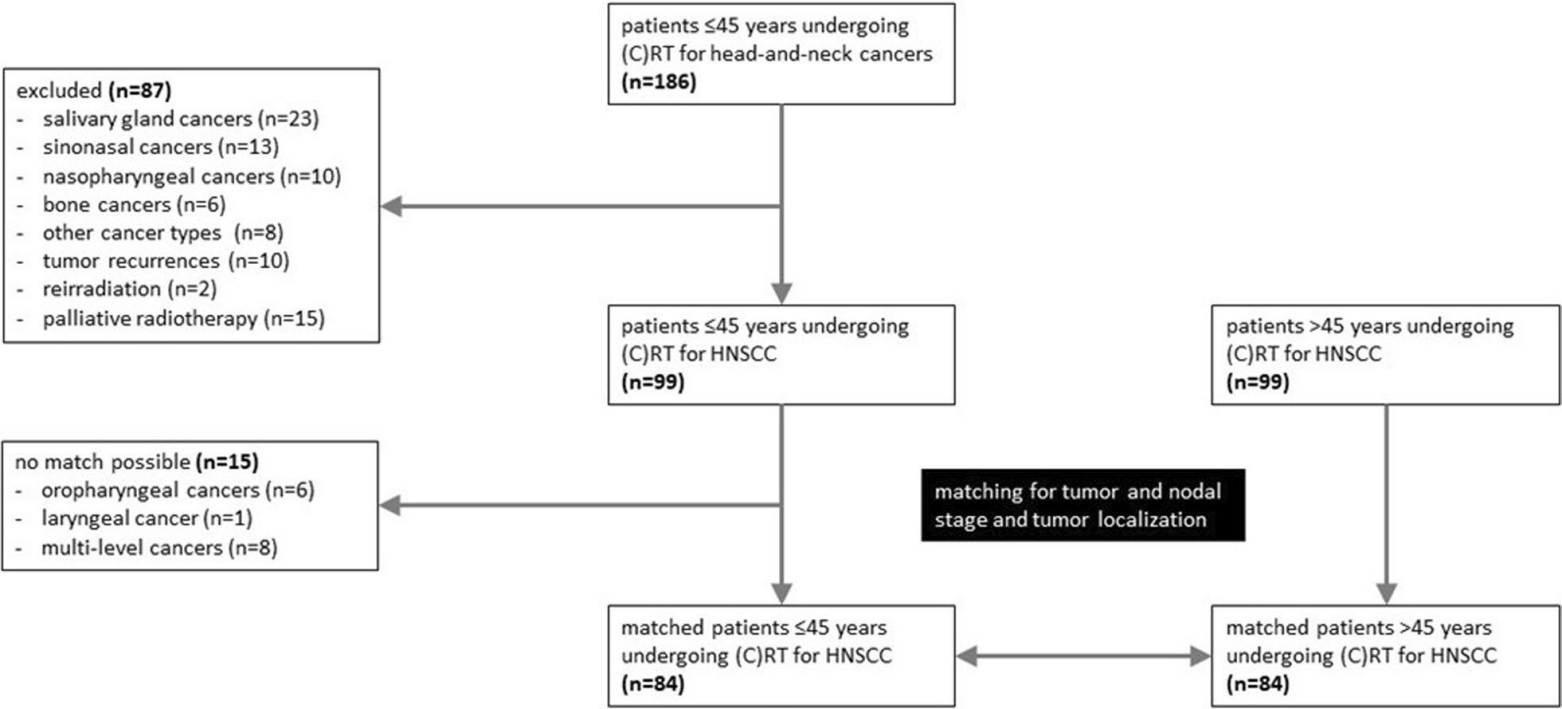
CONSORT diagram for the matched-pair analysis

Patients ≤ 45 years undergoing definitive or adjuvant (chemo)radiation therapy for locoregionally advanced and histologically confirmed HNSCC of the oral cavity, oro-/hypopharynx or larynx between 2009 and 2021 at the Departments of Radiation Oncology of University of Freiburg Medical Center and University of Leipzig Medical Center were retrospectively analyzed. Patients with nasopharyngeal or cutaneous squamous cell cancers, patients exhibiting distant metastases as well as patients treated for tumor recurrence or undergoing re-irradiation were excluded. Details are outlined in the CONSORT diagram (Fig. 1). All patients received treatment based on the recommendations of the institutional multidisciplinary tumor boards.

This analysis was approved in advance by the independent ethics committees of the University of Freiburg (reference no. 389/19) and the University of Leipzig (reference no. 371/23-ek).

### Data collection

Demographic data, clinicopathological parameters and therapy-associated acute and chronic toxicities were collected from the electronic patient records of the respective institutions. Tumor staging was based on the 7th edition of the TNM classification. To compare outcomes of young patients ≤ 45 years with those > 45 years, a bi-institutional control group was matched based on their T and N stages as well as tumor localization. OS was defined from the beginning of (chemo)radiation therapy until death. Progression-free survival (PFS) was calculated from the start of treatment until local/locoregional recurrence, distant progression or death from any cause. Locoregional control (LRC) was defined from the start of (chemo)radiation therapy until first detection of a local recurrence or regional lymph node recurrence/progression. Distant metastasis-free survival (DMFS) was calculated from the start of treatment until the first occurrence of distant metastases or death from any cause. Outcome data not available at the individual centers were supplemented by survival data from the regional cancer registries. Treatment-related acute (≤ 90 days from initiation of radiotherapy) and chronic (> 90 days from initiation of radiotherapy) toxicities were collected based on the Common Terminology Criteria for Adverse Events (CTCAE) version 5. For the calculation of excess mortality rates, survival data were extracted from the registry of the German Federal Statistical Office and matched for patient age and sex as well as year of diagnosis.

### Statistical analyses

Patient data as well as tumor and treatment characteristics were presented as median values with interquartile ranges or frequencies depending on the type of variable. A case–control matching was performed using the tumor extent (T stage), nodal metastases (N stage) and tumor localization as matching variables. The tolerance level for the matching variables was set at 0, resulting in a matched-pair dataset with 84 patients in each age group. Mann–Whitney-U-tests and χ2-tests were used to test for differences between both groups (supplementary Table 1). Survival and control probabilities were determined using the Kaplan–Meier method and compared using log-rank tests. Univariate analyses were performed to assess the influence of clinicopathological variables on patient outcomes, and those parameters with *p* < 0.2 were included in the multivariate analysis based on Cox regression. To address missing data regarding alcohol consumption and smoking, multiple imputation was conducted 13 times, reflecting the proportion of unknown values (13%). The imputed datasets were analyzed separately, and results were pooled to generate robust estimates in univariate analyses. Two-sided *p*-values < 0.05 were considered statistically significant for all analyses. Statistical analyses were performed using SPSS Statistics software, version 25 (IBM, Armonk, NY, USA). The cumulative incidence of local or locoregional failures with death as competing event was calculated using Stata version 18.5 (StataCorp LLC, College Station, TX, USA). Here, the non-parametric Aalen-Johansen estimator (stcompet command in Stata) was used.

## Results

### Clinical and treatment characteristics of young HNSCC patients

After exclusion of 87 patients for divergent histologies, recurrent or metastatic disease or re-irradiation, our cohort comprised 99 patients ≤ 45 years with histologically confirmed HNSCC. Median age was 43 years (18–45 years). The majority of patients (83/99; 83.8%) was male, and most patients exhibited a good performance status (ECOG 0: 17/99; 17.2%; ECOG 1: 45/99; 45.4%). Most patients demonstrated advanced local disease with T3 and T4 cancers diagnosed in 29 (29.3%) and 26% (26.3%) of cases, respectively, and more than half of patients exhibited advanced cervical nodal metastases (N2: 55/99; 55.6%; N3: 7/99; 7.1%). Most cancers were located in the oral cavity (37/99; 37.4%) and oropharynx (32/99; 32.3%). 70.7% of patients (70/99) underwent surgical tumor resection followed by adjuvant radiation or in case of incomplete resection or extranodal spread chemoradiation therapy, and 29.3% (29/99) were treated with primary (chemo)radiotherapy. Chemotherapy was added concurrently to radiotherapy in 58 patients (58.6%). At the time of radiotherapy, 60 patients (60.6%) were active smokers, and 42 patients (42.4%) were consuming alcohol daily. Table 1 provides detailed information about patient and treatment characteristics.

**Table 1:** Characteristics and treatment parameters of patients ≤ 45 years undergoing (C) RT for HNSCC (n = 99)

	n (%)
*Age, years old*	
Median	43
Range	18–45
*Gender*	
Female	16 (16,2)
Male	83 (83,8)
*ECOG*	
0	17 (17.2)
1	45 (45.4)
2	37 (37.4)
*T stage*	
T1	15 (15.2)
T2	28 (28.3)
T3	29 (29.3)
T4	26 (26.3)
Tx	1 (1.0)
*N stage*	
N0	21 (21.2)
N1	16 (16.2)
N2	55 (55.6)
N3	7 (7.1)
*UICC*	
I	8 (8.1)
II	8 (8.1)
III	20 (20.2)
IV	63 (63.6)
*Grading*	
1	3 (3.0)
2	67 (67.7)
3	28 (28.3)
Unknown	1 (1.0)
*Tumor location*	
Oropharynx	32 (32.3)
Hypopharynx	14 (14.1)
Oral cavity	37 (37.4)
Larynx	11 (11.1)
Multilevel	5 (5.1)
*Treatment*	
Surgery + adjuvant (C)RT	70 (70.7)
Definitive (C)RT	29 (29.3)
*Smoking*	
Yes	60 (60.6)
No	28 (28.3)
Unknown	11 (11.1)
*Alcohol*	
Yes	42 (42.4)
No	44 (44.4)
Unknown	13 (13.2)
*Radiotherapy dose (Gy)*	
Median (IQR)	64.0 (60.0–70.0)
*Concomitant chemotherapy*	
Yes	58 (58.6)
No	41 (41.4)

### Oncological outcomes of young HNSCC patients

**Fig. 2:**
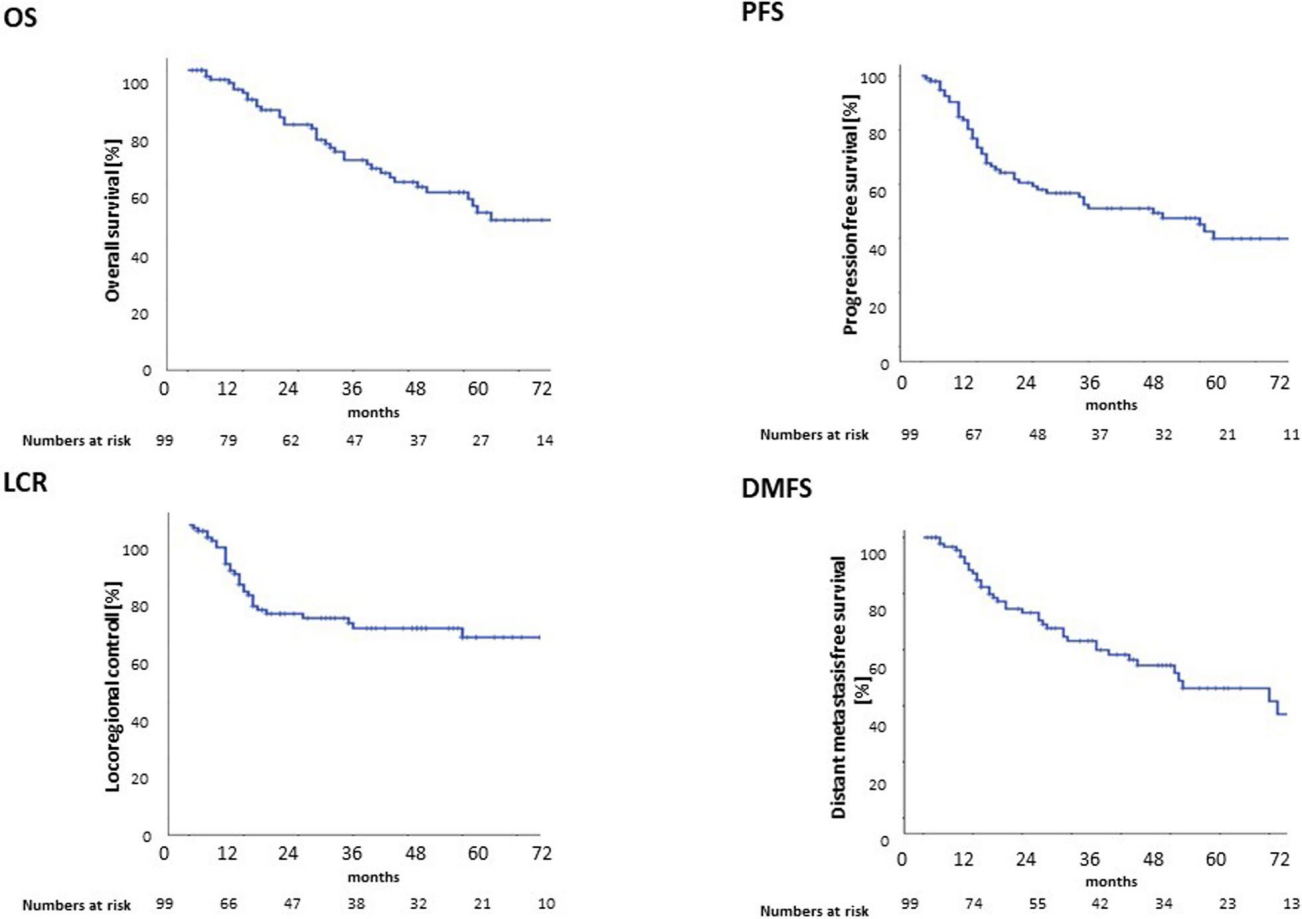
Outcome data for young patients undergoing radiotherapy for HNSCC. Kaplan–Meier curves depict OS (**A**), PFS (**B**), LRC (**C**) and DMFS (**D**) of the whole cohort of patients ≤ 45 years

Median OS of all HNSCC patients ≤ 45 years amounted to 63 months with a median follow up of 59 months, and median PFS and DMFS ranged at 50 and 62 months, respectively (Fig. 2).

**Fig. 3:**
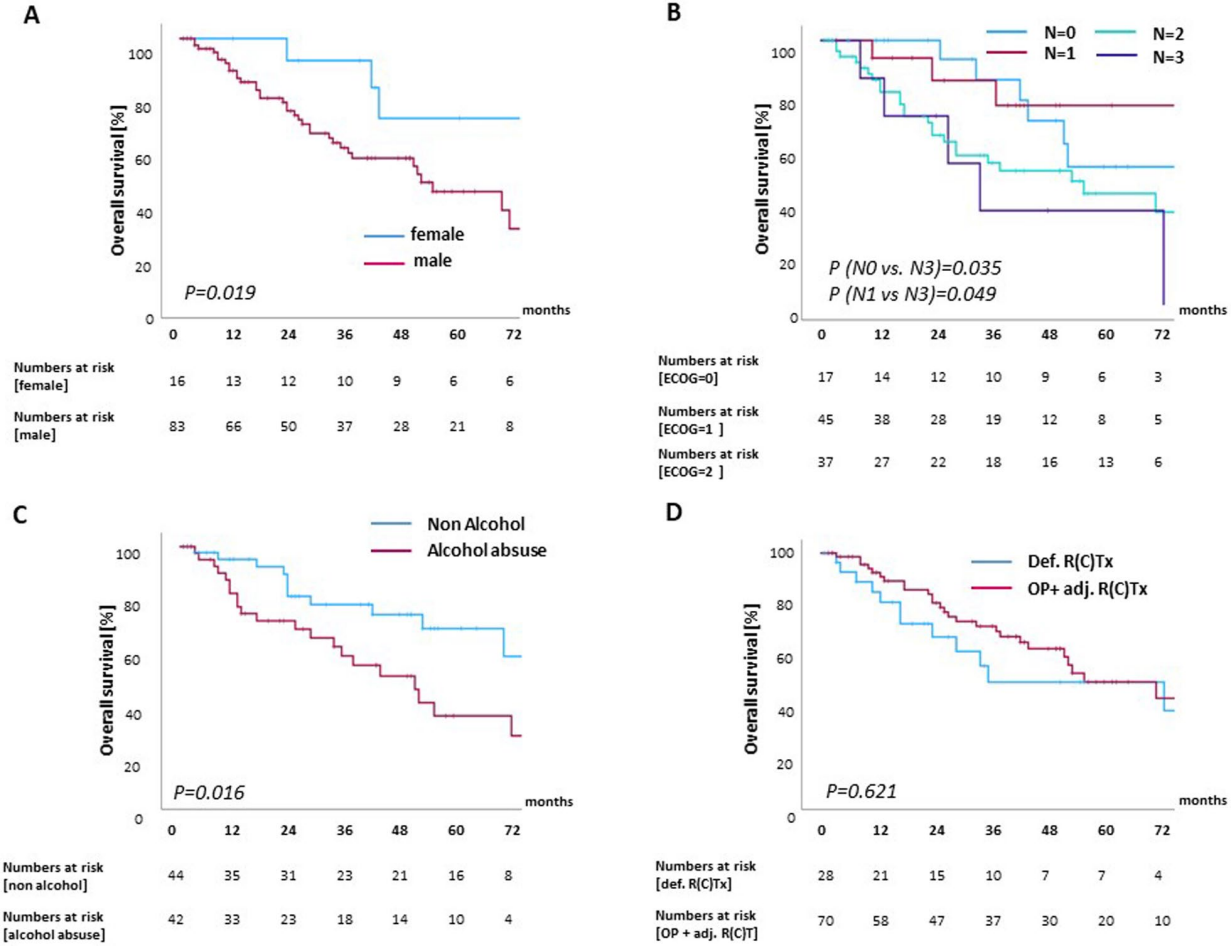
Risk factors for reduced OS in young patients underogoing radiotherapy for HNSCC. Kaplan–Meier curves comparing OS according to sex (**A**), nodal status (**B**), daily alcohol consumption (**C**) and treatment concept (**D**)

1-year, 3-year and 5-year OS probabilities of young HNSCC patients were 93%, 69% and 57%, and 1-year, 3-year and 5-year PFS probabilities were 78%, 53% and 47%, respectively. Most recurrences in the younger HNSCC population occurred within the first year, resulting in 1-year, 3-year and 5-year LRC rates of 80%, 67% and 65%. Using competing risk analysis, the 12-month and 24-month cumulative incidence rates of locoregional recurrence were 19.8% (95% CI, 11.5%–28.0%) and 29.0% (95% CI, 19.5%–38.2%), respectively (supplementary Figure 4). Risk factors for deteriorated OS were analyzed using the Kaplan–Meier method and log-rank tests, with male sex (HR 3.46; 95% CI 1.17–10.23), daily alcohol intake (HR 1.99; 95% CI 0.97–4.07) as well as nodal metastases (HR 1.50; 95%CI 1.05–2.16) significantly impairing OS (Fig. 3). Daily alcohol consumption remained significant risk factors in the multivariate analysis. Similarly, male sex was also associated with decreased PFS (HR 2.74; 95% CI 1.13–6.65) and DMFS (HR 3.61; 95% CI 1.24–10.50), while LRC was not influenced by any patient- or treatment-related variable. Of note, choice of treatment did not influence OS (HR for surgery and adjuvant CRT 0.844; 95% CI 0.43–1.66) or LRC (HR for definitive CRT: 1.53; 95% CI 0.63–3.74) in our cohort. Detailed statistical analyses are summarized in Table 2.

**Table 2:** Uni- and multivariate Cox proportional hazards regression analysis of patient- und tumor-related parameters regarding overall and progression-free survival (n = 99) for patients ≤ 45 years

Overall survival	Univariate			Multivariate		
	HR	95% CI	*p*	HR	95% CI	*p*
Age (continuous)	1.072	0.978–1.175	0.140	1.001	0.907–1.105	0.976
Gender (reference: female)	3.456	1.168–10.230	0.025	2.237	0.689–7.262	0.180
ECOG (continuous)	1.467	0.950–2.265	0.083	1.668	0.976–2.850	0.061
Treatment (reference: definitive radiation)	0.844	0.428–1.661	0.623			
Smoking	1.553	0.723–3.334	0.258			
Alcohol	1.987	0.971–4.069	0.060	**2.522**	**1.172–5.426**	**0.018**
Concomitant systemic treatment	1.193	0.630–2.258	0.588			
Grading	1.307	0.706–2.421	0.394			
T status	1.211	0.888–1.652	0.227			
N status	1.503	1.046–2.160	0.028	1.266	0.807–1.986	0.305

Progression-free survival	Univariate			Multivariate		
	HR	95% CI	*p*	HR	95% CI	*p*
Age (continuous)	1.012	0.941–1.088	0.749			
Gender (reference: female)	2.740	1.129–6.649	0.026			
ECOG (continuous)	1.010	0.693–1.474	0.957			
Treatment (reference: definitive radiation)	1.102	0.575–2.110	0.770			
Smoking	1.200	0.639–2.252	0.571			
Alcohol	1.355	0.762–2.410	0.301			
Concomitant systemic treatment	1.181	0.677–2.060	0.559			
Grading	1.307	0.757–2.256	0.337			
T status	1.272	0.968–1.671	0.084			
N status	1.161	0.858–1.570	0.333			

Locoregional control	Univariate			Multivariate		
	HR	95% CI	*p*	HR	95% CI	*p*
Age (continuous)	0.988	0.909–1.073	0.767			
Gender (reference: female)	2.700	0.805–9.055	0.108	2.869	0.848–9.710	0.090
ECOG (continuous)	0.679	0.422–1.092	0.110	0.654	0.402–1.061	0.086
Treatment (reference: definitive radiation)	1.531	0.627–3.741	0.350			
Smoking	0.991	0.457–2.146	0.981			
Alcohol	0.953	0.459–1.976	0.896			
Concomitant systemic treatment	1.575	0.758–3.272	0.224			
Grading	1.055	0.529–2.104	0.880			
T status	1.165	0.823–1.649	0.389			
N status	0.998	0.686–1.453	0.993			

Distant metastasis free survival	Univariate			Multivariate		
	HR	95% CI	*p*	HR	95% CI	*p*
Age (continuous)	1.055	0.959–1.161	0.273			
Gender (reference: female)	3.601	1.235–10.498	0.019	2.219	0.692–7.120	0.180
ECOG (continuous)	1.187	0.768–1.835	0.440			
Primary treatment (reference: definitive radiation)	0.765	0.390–1.499	0.435			
Smoking	1.375	0.646–2.923	0.407			
Alcohol	1.803	0.881–3.689	0.106	1.694	0.862–3.329	0.126
Concomitant systemic treatment	1.258	0.674–2.349	0.472			
Grading	1.636	0.872–3.069	0.125	1.616	0.843–3.099	0.149
T status	1.245	0.914–1.697	0.165	1.076	0.776–1.494	0.660
N status	1.476	1.037–2.102	0.031	1.242	0.834–1.848	0.286

Statistically significant values printed in bold. CI, confidence interval; ECOG, eastern cooperative oncology group; HR, hazard ratio; UICC, union for international cancer control

### *Outcome comparison of young patients with patients *> *45 years*

**Fig. 4:**
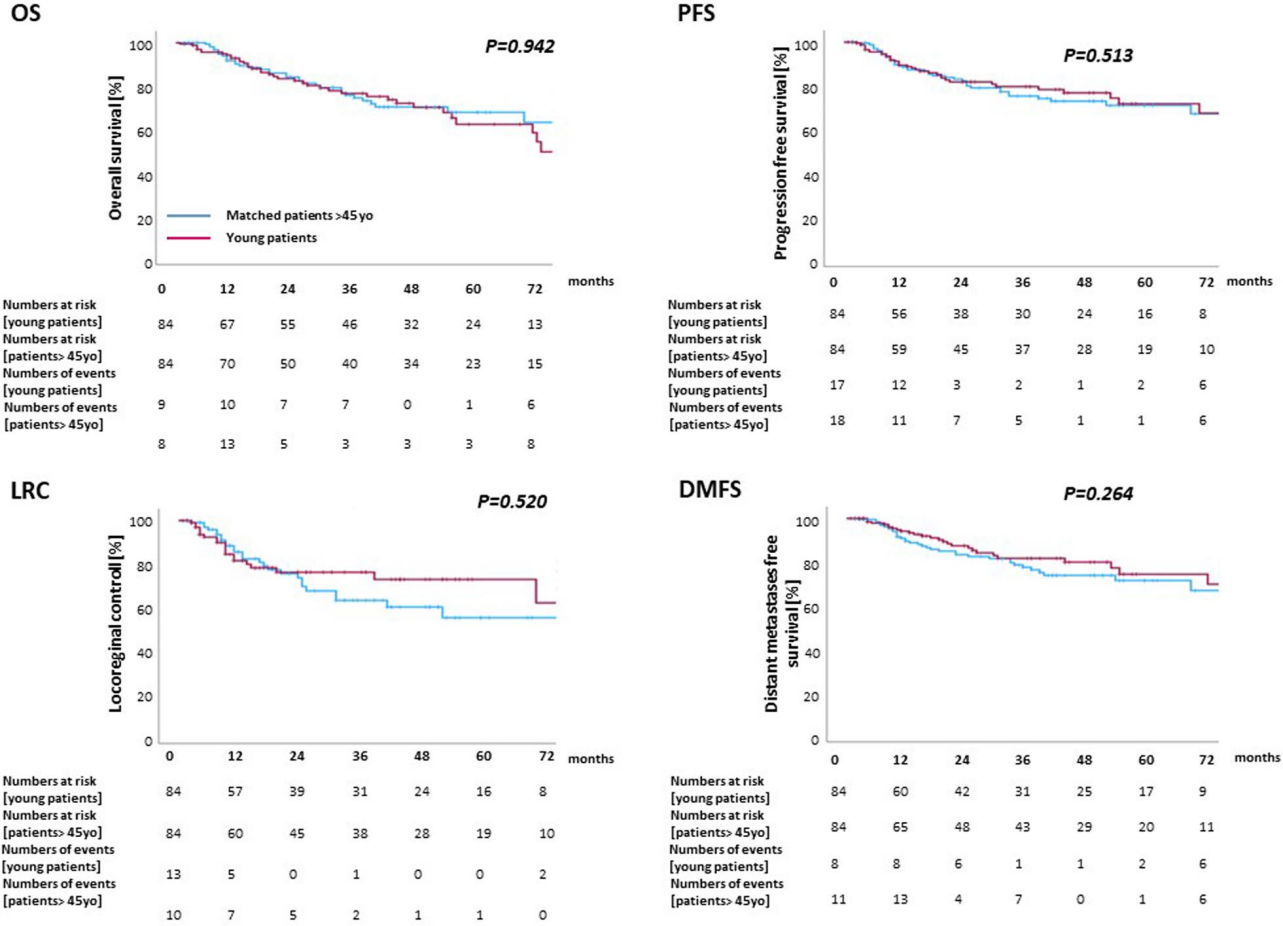
Comparison of oncological outcomes between patients ≤ 45 years and patients > 45 years after matching for tumor stage, nodal stage and tumor localization. Kaplan–Meier curves depict OS (**A**), PFS (**B**), LRC (**C**) and DMFS (**D**)

Survival outcomes of patients ≤ 45 years and those > 45 years were compared by a matched-pair analysis. Matching was performed for T and N categories and tumor localization, and 84 patients could be included in each group. Details of both cohorts are outlined in supplementary Table 1. There was no significant difference in OS between patients ≤ 45 years and those > 45 years (p = 0.94), with a median OS of 65 months for patients ≤ 45 years (95% CI 34.8–95.2) vs. 82 months (95% CI 37.8–126.2). Similarly, there was no difference in LRC (*p* = 0.52), PFS (median PFS 52 months [95% CI 23.9–80.1] vs. 37 months [95% CI 20.5–53.5], *p* = 0.51), or DMFS (median DMFS 86 months [95% CI 37.4–134.6] vs. 64 months [95% CI 26.7–101.3], *p* = 0.26) (Fig. 4). 1-year and 3-year OS probabilities amounted to 90% and 65% for patients ≤ 45 years and 89% and 66% for those > 45 years, respectively.

However, the sensitivity analysis to assess HNSCC-related excess mortality as compared to the mean life expectancy of the German general population as corrected for sex and age at the time of diagnosis revealed significantly increased mortality in HNSCC patients of age ≤ 45 years. While the excess mortality in patients > 45 years was above 5.9-fold, (HR 5.896; 95%-CI 3.28–10.60), younger patients ≤ 45 years had a 59-fold excess mortality rate compared to the life expectancy of the sex- and age-matched German general population (HR 58.71; 95%-CI 14.03–245.8).

With the majority of tumors in the young cohort localized to the oral cavity, where HPV status does not play a significant role, we conducted a subgroup survival analysis (Supplementary Figure 2). While OS, PFS, and DMFS did not differ significantly between age groups, LRC was significantly lower in younger patients (*p* = 0.036). Tumors with other localizations showed no significant differences in survival outcomes across age groups. ((Supplementary Figure 3).

### Radiotherapy-associated toxicity patterns in young HNSCC patients

Radiotherapy-related toxicities were categorized according to the Common Terminology Criteria for Adverse Events (CTCAE, version 5) and were divided between acute and chronic toxicities. Mucositis was observed as the most common treatment-associated acute toxicity with 56 (57.7%) and 38 (39.2%) patients exhibiting mild/moderate and higher-grade mucositis, respectively. Higher-grade dysphagia was observed in 33 patients (34%), and 34 patients (35.1%) suffered from higher-grade acute pain. Higher-grade chronic toxicities were rare, with higher-grade lymphedema, cervical fibrosis, or xerostomia reported by 2 patients (2.1%) each. Only higher-grade dysphagia exhibited a higher prevalence with 8 patients (8.2%) suffering from persistent feeding tube dependency. No fatal toxicity was observed in our patient cohort. Detailed toxicity data are presented in Table 3.

**Table 3:** Treatment-associated toxicities of patients ≤ 45 years (n = 97)

	CTCAE 0	CTCAE 1/2	CTCAE 3/4	CTCAE 5
**Acute toxicity**	n = 97			
Mucositis	3 (3.1)	56 (57.7)	38 (39.2)	0
**Dermatitis**	**2 (2.1)**	**76 (76.8)**	**19 (19.6)**	**0**
Dysphagia	12 (12.4)	52 (53.6)	33 (34.0)	0
Infection	78 (80.4)	18 (18.6)	1 (1.0)	0
Pain	14 (14.4)	49 (50.5)	34 (35.1)	0
*Chronic toxicity*				
Lymphedema	71 (73.2)	24 (24.7)	2 (2.1)	0
Fibrosis	75 (77.3)	20 (22.2)	2 (2.1)	0
Osteonecrosis	88 (90.7)	4 (4.1)	5 (5.2)	0
Xerostomia	69 (71.1)	26 (26.8)	2 (2.1)	0
Dysphagia	66 (68.0)	23 (23.7)	8 (8.2)	0
Dysgeusia	67 (69.1)	30 (30.9)	0	0
Fistula	93 (95.9)	4 (4.1)	0	0

## Discussion

In this large bi-institutional study including a total of 99 young patients with HNSCC undergoing (chemo)radiation, male sex was identified as the key risk factor for reduced PFS and DMFS, while alcohol consumption was found to be associated with diminished OS. In the matched-pair analysis comparing with patients aged > 45 years, there was no difference for OS, PFS, LRC, and DMFS. Chronic radiotherapy-induced higher-grade chronic toxicities were rare among young adults with HNSCC.

There are to some extent controversies whether young adults with HNSCC have differences in their oncological outcomes compared to older patients. In a retrospective analysis from the Icahn School of Medicine comparing the outcomes between young (≤ 45 years) and older (> 45 years) adults with squamous cell carcinoma of the oral tongue, the younger population exhibited significantly lower rates of 5-year LRC (79.6% vs. 52.5%, *p* = 0.043) and 5-year DMFS (88.1% vs. 61.8%, *p* = 0.006), whereas 5-year OS (55.5% vs. 58.1%) was comparable [12]. In contrast, Lacy et al. observed that young HNSCC patients (≤ 40 years) had significantly better 5-year OS rates than middle-aged and older patients (65% young patients vs. 52% middle-aged patients vs. 38% old patients), even after controlling for other factors such as smoking, comorbidities, primary tumor site, tumor stage, and nodal disease [21]. Another retrospective study including 284 patients with oral cavity cancer also compared oncological outcomes between young (< 45 years) and older (55–70 years) adults [22]. Here, neither OS nor disease-specific survival were different between both groups after matching by sex and stage. In a large Canadian retrospective analysis with 185 young HNSCC patients (< 40 years), oncological outcomes were compared with a corresponding cohort of older HNSCC patients that was matched for site, sex, and date of presentation. Here, OS was significantly higher in the young cohort (5-year OS 68% vs. 49%, *p* = 0.001), whereas cause-specific survival was not statistically different between the two groups [23]. In line with this finding, Dougherty et al. found improved OS but similar disease-free survival in 59 young HNSCC patients compared to a corresponding older HNSCC cohort [24]. Even though there are conflicting results, the results of our analysis and the majority of previous studies suggest that OS may rather be (slightly) higher in the young HNSCC population, potentially related to fewer comorbidities and the lower risk of dying from non-cancer related deaths [25, 26]. However, compared to the average life expectancy of the German general population according to sex and age, we found a considerably higher, 59-fold excess mortality of in young HNSCC patients ≤ 45 years, while patients ≥ 45 years only exhibited a sixfold excess mortality. Indeed, the OS in young patients ≤ 45 years appears to be substantially diminished in light of a life expectancy of 39.02 (95%-CI 37.91–40.13) years versus 20.55 (95%-CI 19.05–22.05) years in the matched general population despite reduced exposure to various environmental, occupational and life style-related risk factors.

While smoking is a well-known risk factor for worse prognosis after (chemo)radiation in the general HNSCC population [27, 28], it had no prognostic value in our cohort. In a large analysis from 2073 patients with oral cavity squamous cell carcinoma (OSsCC) including 9% young patients (≤ 40 years), individuals were categorized by age and smoking status [29]. Young non-smokers with OSCC showed higher mortality compared to young smokers, driven by increased regional and distant recurrences. Young non-smokers with OSCC also exhibited higher neutrophil-to-lymphocyte ratios compared to controls, suggesting immune dysfunction may contribute to their poorer prognosis. However, there are also reports that reported opposite findings: Révész et al. identified non-smoking as favorable prognostic factor in the univariate analysis within a cohort of young HNSCC patients [8]. Another retrospective analysis in which 78 young (< 40 years) HNSCC patients were analyzed, showed that never-smokers/never-drinkers were diagnosed at a younger median age (31.5 years vs. 35.5 years, *p* = 0.007). They were also more likely to be female (75% vs. 30%, *p* < 0.001), they had a higher incidence of OSCCs (57% vs. 24%, *p* = 0.003) and T1 disease (47% vs. 20%, *p* = 0.01) [30]. There was no significant difference in 10-year relapse-free survival between never-smokers/never-drinkers and smokers and/or drinkers, but there was a trend towards improved 10-year OS for the former (71% vs. 46%, *p* = 0.10). Based on these controversial findings, there is a need for further multi-center and prospective studies in which prognostic factors of young HNSCC patients are investigated, preferentially independently for the different subsites of HNSCC.

Daily alcohol consumption was identified as a risk factor for reduced OS both in the univariate and the multivariate analysis of our study, although due to the retrospective nature of our dataset, detailed information on the exact amount of alcohol intake could not be elucidated.. A previous study with 85 young (< 40 years) adults with HNSCC also observed a negative prognostic value of alcohol intake regarding OS; alcohol consumption remained a significant prognosticator also in the multivariate analysis. Several high-quality cohort studies have demonstrated a relationship between alcohol consumption and impaired OS in the general population of HNSCC patients [31–34], although other analyses could not confirm an association after controlling for other prognostic factors [35, 36]. Only one randomized controlled trial of a treatment intervention for harmful alcohol use in HNSCC patients has been conducted yet, and this trial could not demonstrate significantly lower rates of alcohol intake in the intervention group in which cognitive behavioral therapy was applied [37]. However, guidelines recommend encouraging patients to modify excessive alcohol consumption and referring patients for alcohol cessation counseling if indicated. [38–40].

Given the long life expectancy that surviving young HNSCC patients commonly have, there is a strong need to thoroughly assess chronic radiotherapy-related toxicities in this cohort. In our analysis, long-term feeding tube dependency was moderate with 8.2%. In a small study evaluating radiotherapy-related toxicities in 22 young head-and-neck cancer patients and comparing toxicity rates with an older head-and-neck cancer cohort, all with similar tumor stages and treatment protocols, there were no significant differences between the groups regarding the incidences or severity of xerostomia, dysphagia, dysgeusia, and radiodermatitis [41]. Interestingly, young patients showed a higher incidence and severity of oral mucositis and trismus compared to older patients [42]. However, most publications analyzing young adults with HNSCC did not report any data on radiotherapy-related sequelae [23, 43, 44]. Therefore, both physician-assessed and patient-reported outcomes should be analyzed in further studies to assess the impact of radio- and chemotherapy on the quality-of-life of surviving young adults with HNSCC.

In our study, 71% of patients in the young cohort underwent primary surgery, compared with 51% in the matched cohort of older patients. Although we made considerable efforts to adjust for life expectancy and other factors, these adjustments may not fully eliminate potential confounding biases, such as fewer comorbidities or more aggressive salvage therapies in the event of recurrence or metastasis (23.8% in the cohort ≤ 45 years vs. 15.5% patients > 45 years old). Nevertheless, the observed differences in treatment patterns reflect real-world clinical practice, where treatment decisions are influenced by a variety of patient-specific factors, including age, comorbidities, and overall life expectancy.

Though presenting one of the largest and most homogenous datasets reporting special characteristics and prognostic parameters in young adults with HNSCC undergoing (chemo)radiation, there are some limitations of our study which are mainly related to the retrospective nature of the analysis. Considering the retrospective assessment of radiotherapy-related toxicities and the known risk of underestimating toxicities through this approach, the observed rates of acute and chronic toxicities should be considered with caution. As we included patients treated with (chemo)radiation since 2009, the majority of patients had no information on their HPV status available so that this parameter could not be incorporated into our analysis. In addition, the exact cause of death was not known for all patients, therefore not allowing us to analyze disease-specific survival in our cohort.

## Conclusions

In conclusion, our analysis indicates that young HNSCC patients (≤ 45 years) treated with (chemo)radiation demonstrate similar OS, PFS, LRC, and DMFS outcomes compared to older patients, with no significant differences observed between the two age groups. Male gender, daily alcohol intake and nodal metastases were identified as potential risk factors for impaired survival in the younger cohort. Even though we observed low rates of chronic radiotherapy-induced toxicities among young adults with HNSCC, there is a strong need for further prospective studies regarding the long-term impact of (chemo)radiation on patient-reported quality-of-life.

## Supplementary Information

Supplementary file 1.

Supplementary file 2.

## Data Availability

Data available from the authors upon reasonable request.
