# Supplementary material for: Radiotherapy outcomes and risk factors for young patients with head-and-neck squamous cell carcinomas: a matched-pair analysis
Source: Radiat Oncol. 2025 Apr 22;20:62. doi: 10.1186/s13014-025-02631-w (PMC12016063; doi:10.1186/s13014-025-02631-w)
Supplement: Supplementary file 1 [file 13014_2025_2631_MOESM1_ESM.pdf]

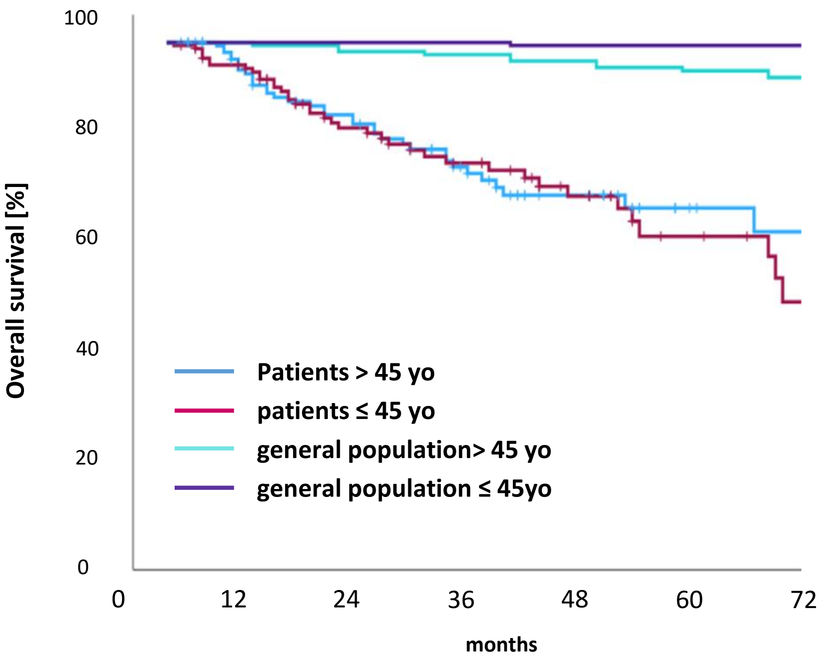

**Numbers at risk [patients > 45 yo]**	84	67	55	46	32	24	13	10
**Numbers at risk [patients ≤ 45yo]**	84	70	50	40	34	23	15	13
**Numbers at risk [population> 45 yo]**	84	84	83	81	80	78	76	75
**Numbers at risk [population ≤ 45 yo]**	84	84	84	84	84	83	83	83

Figure 1 Supplement:
Overall survival for gematched patients elder than 45 years old, young patients under 45 years old as well as expected life expancy for each group

OS

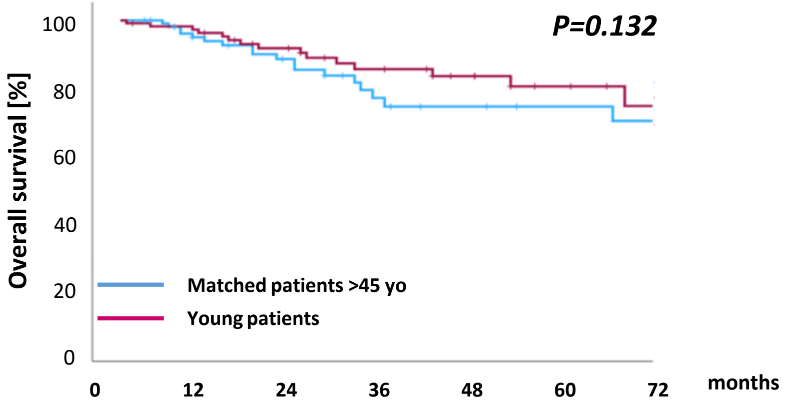

	0	12	24	36	48	60	72
Numbers at risk [young patients]	37	34	25	20	17	11	8
Numbers at risk [patients> 45yo]	37	30	23	17	11	10	8
Numbers of events [young patients]	2	6	2	2	1	1	2
Numbers of events [patients> 45yo]	4	5	4	4	0	0	5

PFS

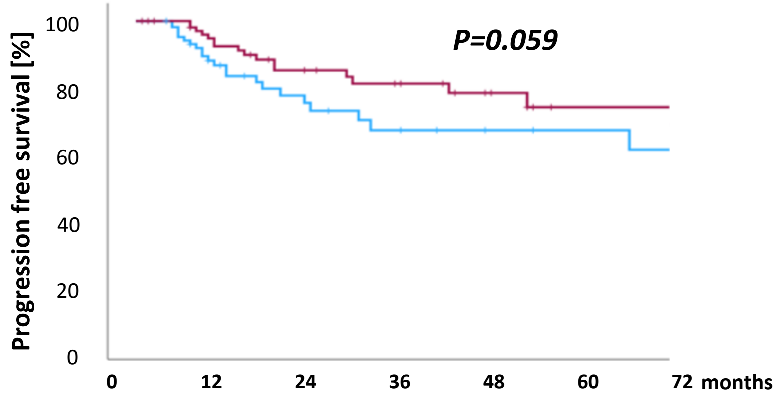

	0	12	24	36	48	60	72
Numbers at risk [young patients]	37	29	19	16	13	8	4
Numbers at risk [patients> 45yo]	37	25	16	12	9	7	6
Numbers of events [young patients]	4	8	1	1	1	1	2
Numbers of events [patients> 45yo]	10	6	3	2	0	0	5

LRC

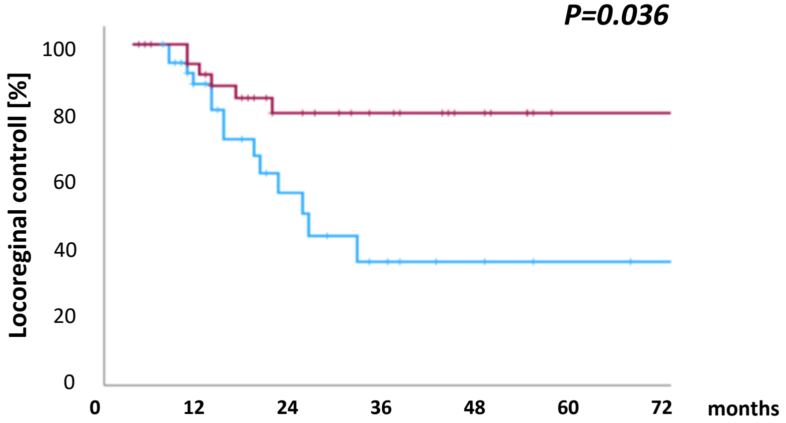

	0	12	24	36	48	60	72
Numbers at risk [young patients]	37	30	20	17	13	8	4
Numbers at risk [patients> 45yo]	37	27	17	13	9	7	6
Numbers of events [young patients]	3	3	0	0	0	0	1
Numbers of events [patients> 45yo]	4	6	3	1	0	0	0

DMFS

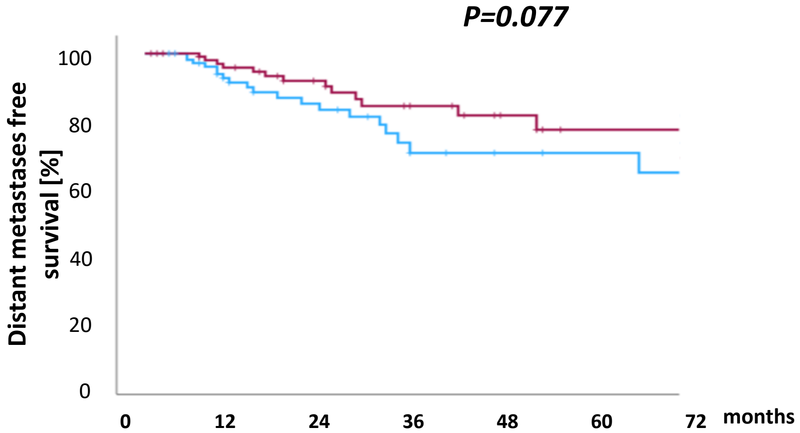

	0	12	24	36	48	60	72
Numbers at risk [young patients]	37	30	21	16	13	8	4
Numbers at risk [patients> 45yo]	37	30	19	15	9	7	6
Numbers of events [young patients]	2	5	3	1	1	1	1
Numbers of events [patients> 45yo]	4	7	3	4	0	0	5

Figure 2 supplement:
OS, PFS, LRC and DMFS for gematched coohrte **with oral cavity carcinoma**, young patients ≤ 45 yo vs. control group

OS

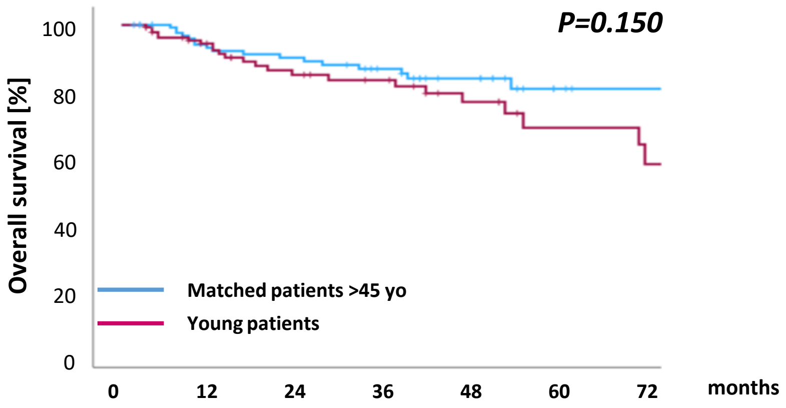

	0	12	24	36	48	60	72
Numbers at risk [young patients]	47	36	25	20	17	12	7
Numbers at risk [patients> 45yo]	47	37	32	29	21	14	5
Numbers of events [young patients]	6	7	3	1	2	2	6
Numbers of events [patients> 45yo]	5	5	3	3	0	1	1

PFS

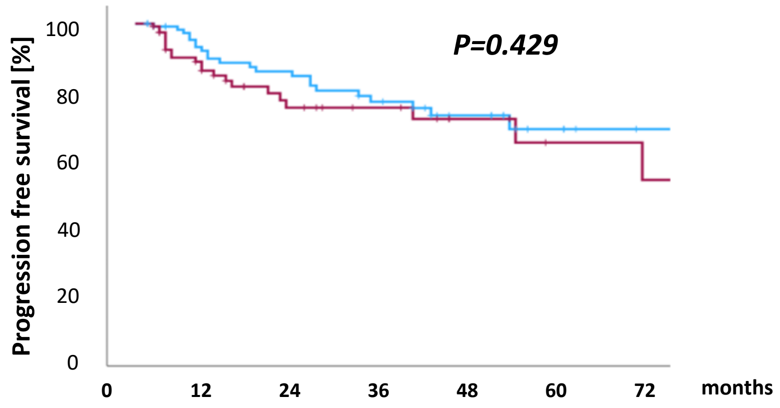

	0	12	24	36	48	60	72
Numbers at risk [young patients]	47	27	19	14	11	8	4
Numbers at risk [patients> 45yo]	47	34	29	25	19	12	4
Numbers of events [young patients]	13	4	2	1	0	1	4
Numbers of events [patients> 45yo]	8	5	4	3	1	1	1

LRC

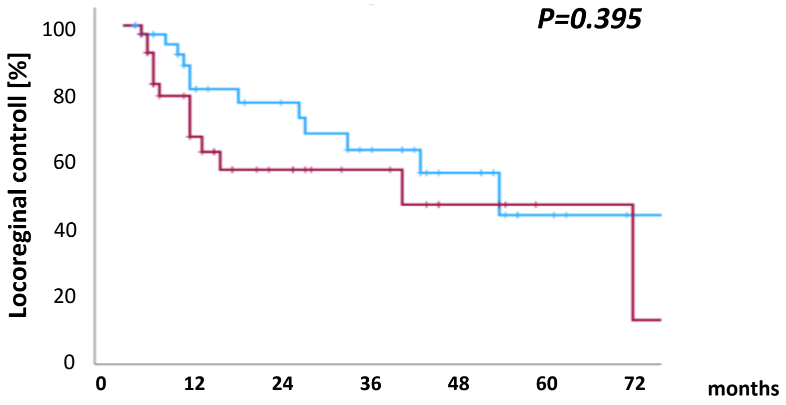

	0	12	24	36	48	60	72
Numbers at risk [young patients]	47	27	19	14	11	8	4
Numbers at risk [patients> 45yo]	47	33	28	25	19	12	4
Numbers of events [young patients]	10	2	0	1	0	0	1
Numbers of events [patients> 45yo]	6	1	2	1	1	1	0

DMFS

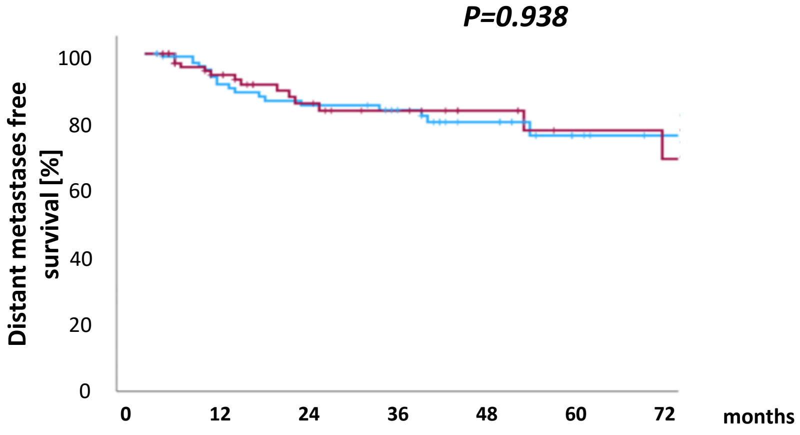

	0	12	24	36	48	60	72
Numbers at risk [young patients]	47	30	21	15	12	9	5
Numbers at risk [patients> 45yo]	47	35	29	28	20	13	5
Numbers of events [young patients]	6	3	3	0	0	1	5
Numbers of events [patients> 45yo]	7	6	1	3	0	1	1

Figure 3 supplement:
OS, PFS, LRC and DMFS for gematched coohrte **with other tumor locations excpet of oral cavity carcinoma**,
young patients ≤ 45 yo vs. control group matched patients > 45 yo.

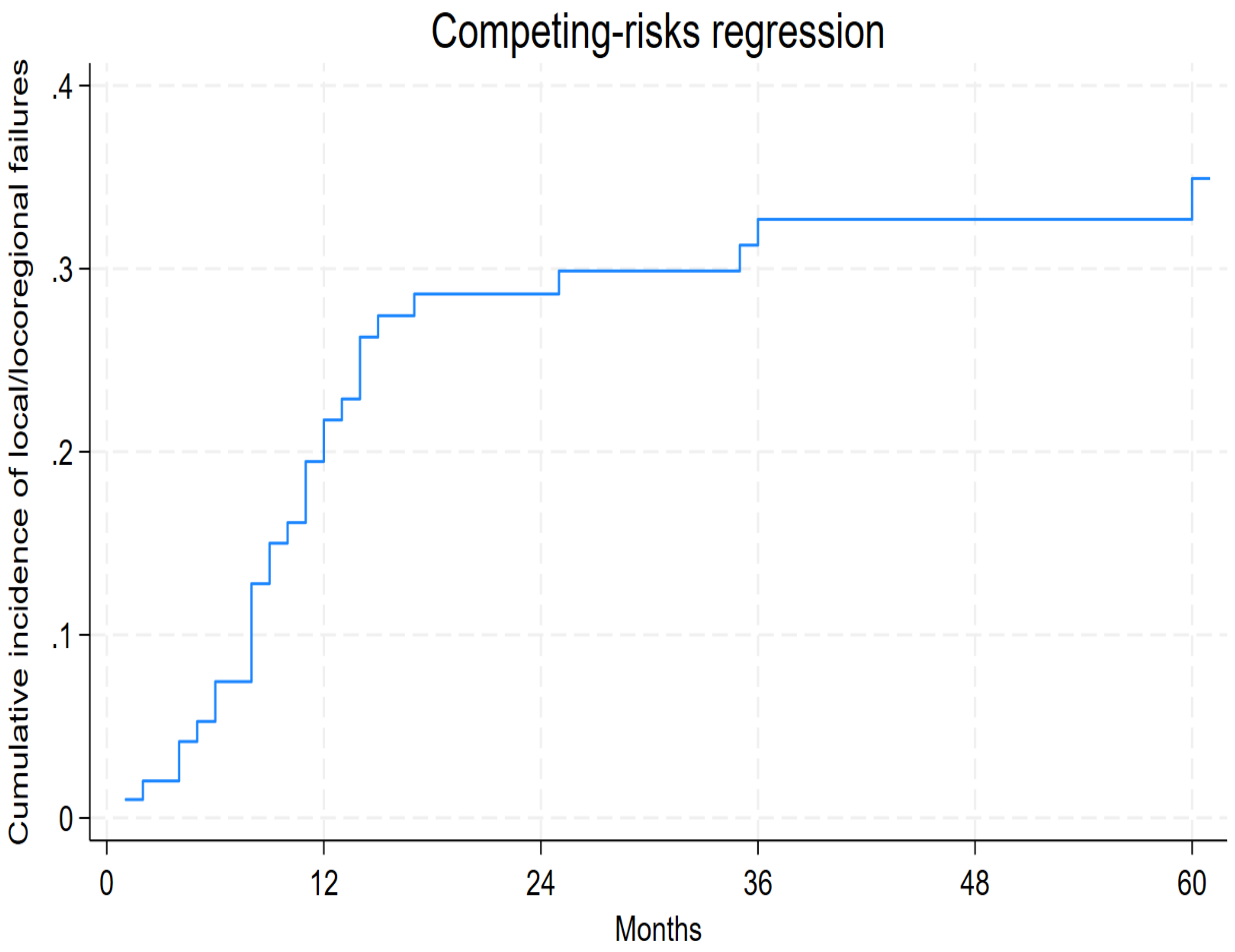

Figure 4 supplement:
cumulative incidence of local/locoregional recurrences, with death as a competing event, for patients ≤ 45 years old
