# Supplementary material for: Radiotherapy outcomes and risk factors for young patients with head-and-neck squamous cell carcinomas: a matched-pair analysis
Source: Radiat Oncol. 2025 Apr 22;20:62. doi: 10.1186/s13014-025-02631-w (PMC12016063; doi:10.1186/s13014-025-02631-w)
Supplement: Supplementary file 2 [file 13014_2025_2631_MOESM2_ESM.docx]

**Supplementary table 1:**

**Pair-matched characteristics and treatment baseline of patients ≤45 years and >45 years (n=84). The 7^th^ edition of the TNM classification was used. Groups were compared using Mann-Whitney-U-Test (radiation dosis) and χ2-tests.**

ECOG=Eastern Cooperative Oncology Group. (C)RT= (chemo)radiotherapy.

|  | **Young patients ≤45 yo** | **Matched Cohort**  **Patients > 45 yo** |  |
| --- | --- | --- | --- |
|  | **N=84 (%)** | **N=84 (%)** | **p-value** |
| **Age, years old**  **Median (IQR)**  **Range** | 43 (40-44)  18-45 | 62 (56-68)  47-84 |  |
| **Gender**  Female  Male | 15 (17.9)  69 (82.1) | 18 (21.4)  66 (78.6) | 0.560 |
| **ECOG**  0  1  2  3 | 12 (14.3)  35 (41.7)  37 (44.0)  0 | 15 (17.8)  39 (46.4)  28 (33.3)  2 (2.4) | 0.284 |
| **T stage**  T1  T2  T3  T4  Tx | 14 (16.7)  28 (33.3)  25 (29.8)  16 (19.0)  1 (1.2) | 14 (16.7)  28 (33.3)  25 (29.8)  16 (19.0)  1 (1.2) | 1.00 |
| **N stage**  N0  N1  N2  N3 | 17 (20.2)  15 (17.9)  47 (56.0)  5 (6.0) | 17 (20.2)  15 (17.9)  47 (56.0)  5 (6.0) | 1.00 |
| **UICC**  I  II  III  IV | 5 (6.0)  9 (10.7)  16 (19.0)  54 (64.3) | 5 (6.0)  9 (10.7)  16 (19.0)  54 (64.3) | 1.00 |
| **Grading**  1  2  3  Unknown | 3 (3.6)  57 (67.9)  23 (27.4)  1 (1.2) | 4 (4.8)  55 (65.5)  24 (28.6)  1 (1.2) | 0.754 |
| **Tumor location**  Oropharynx  Hypopharynx  Oral cavity  Larynx | 26 (31.0)  14 (16.7)  37 (44.0)  7 (8.3) | 26 (31.0)  14 (16.7)  37 (44.0)  7 (8.3) | 1.00 |
| **Chemotherapy**  No concomitant chemotherapy  Concomitant chemotherapy | 28 (30.8)  56 (69.2) | 29 (30.8)  55 (69.2) | 0.871 |
| **Radiotherapy dose (Gy)**  Median (IQR)  Range | 64.0 (60.0-69.3)  55.0 – 72.0 | 66.0 (61.9-70.0)  50.0-74.0 | 0.083 |
| **Treatment**  Surgery+ adjuvant (C)RT  Definitive (C)RT | 62(71.4)  22(28.6) | 42 (50.0)  42 (50.0) | **0.001** |
| **Smoking**  Yes  No  Unknown | 50 (5.,5)  28 (33.3)  6 (7.1) | 47 (56.0)  17 (20.2)  20 (23.8) | 0.234 |
| **Alcohol**  Yes  No  Unknown | 35 (35.7)  43 (47.6)  6 (16.7) | 18 (21.4)  46 (56.0)  20 (22.6) | **0.040** |
| **Salvage therapy**  Surgery  Palliative system therapy  Re-radiation | 7 (8.33)  8 (9.52)  5 (5.95) | 5 (5.95)  7 (8.33)  1 (1.19) | 0.228 |

**Supplementary table 2: HPV status of young patients with HNSCC and oropharynx carcinoma (OPC).**

|  | | HPV status of young patients with HNSCC | HPV status of young patients with OPC |  |
| --- | --- | --- | --- | --- |
|  | | N=99 (%) | N=32 (%) |  |
|  | neg | 21 (21.2) | 4(12.5) |  |
|  | pos | 8 (8.1) | 7(21.9) |  |
|  | unknown | 70 (70.7) | 21(65.6) |  |
|  | | | | |
|  | | | | |
